# Profiles of Endogenous Phytohormones Over the Course of Norway Spruce Somatic Embryogenesis

**DOI:** 10.3389/fpls.2018.01283

**Published:** 2018-09-06

**Authors:** Zuzana Vondrakova, Petre I. Dobrev, Bedrich Pesek, Lucie Fischerova, Martin Vagner, Vaclav Motyka

**Affiliations:** ^1^Laboratory of Biologically Active Compounds, Institute of Experimental Botany of the Czech Academy of Sciences, Prague, Czechia; ^2^Laboratory of Hormonal Regulations in Plants, Institute of Experimental Botany of the Czech Academy of Sciences, Prague, Czechia; ^3^Laboratory of Mass Spectrometry, Institute of Experimental Botany of the Czech Academy of Sciences, Prague, Czechia

**Keywords:** somatic embryos, auxins, cytokinins, abscisic acid, jasmonates, salicylic acid, plant growth regulators, *Picea abies*

## Abstract

Conifer somatic embryogenesis (SE) is a process driven by exogenously supplied plant growth regulators (PGRs). Exogenous PGRs and endogenous phytohormones trigger particular ontogenetic events. Complex mechanisms involving a number of endogenous phytohormones control the differentiation of cells and tissues, as well as the establishment of structures and organs. Most of the mechanisms and hormonal functions in the SE of conifers have not yet been described. With the aim to better understand these mechanisms, we provided detailed analysis of the spectrum of endogenous phytohormones over the course of SE in Norway spruce *(Picea abies)*. Concentrations of endogenous phytohormones including auxins, cytokinins (CKs), abscisic acid (ABA), jasmonates, and salicylic acid (SA) in somatic *P. abies* embryos were analyzed by HPLC-ESI-MS/MS. The results revealed that the concentrations of particular phytohormone classes varied substantially between proliferation, maturation, desiccation, and germination. Endogenous ABA showed a maximum concentration at the maturation stage, which reflected the presence of exogenous ABA in the medium and demonstrated its efficient perception by the embryos as a prerequisite for their further development. Auxins also had concentration maxima at the maturation stage, suggesting a role in embryo polarization. Endogenous jasmonates were detected in conifer somatic embryos for the first time, and reached maxima at germination. According to our knowledge, we have presented evidence for the involvement of the non-indole auxin phenylacetic acid, *cis*-zeatin- and dihydrozeatin-type CKs and SA in SE for the first time. The presented results represent the currently most comprehensive overview of plant hormone levels in embryos throughout the whole process of conifer SE. The differences in concentrations of various classes of phytohormones over the proliferation, maturation, desiccation, and germination in somatic *P. abies* embryos clearly indicate correlations between endogenous phytohormone profiles and particular developmental stages of the SE of conifers.

## Introduction

Somatic embryogenesis is a five-step developmental process during which somatic cells dedifferentiate and divide to initiate embryogenic development. Under suitable conditions, the early embryo can mature and even recreate a whole plant. The first reports of conifer SE from *Picea abies* seed explants were published in 1985 (Hakman et al., 1985; Chalupa, 1985). Since then, embryogenic cultures have been induced in many conifer species and different cell lines. Propagation through SE not only enables the formation of multiple genetically identical embryos, but also offers researchers a suitable system for studying embryonic development and regulation in detail (von Aderkas and Bonga, 2000; Cairney and Pullman, 2007).

The protocol for successful SE induction varies among plants. Although most plants require similar physical conditions (temperature, light regime) for the induction of SE, medium composition can have a large impact on SE outcome. Changes in media constituents, especially in exogenously applied PGRs, are decisive for the progression of embryos through the steps of SE (von Arnold et al., 2002). Embryogenic cultures are usually induced using media supplemented with CKs, while proliferation proceeds with media that contain either CKs alone or in combination with auxins. ABA is necessary during the maturation process, whereas desiccation and germination represent the PGR-independent steps of SE (von Arnold et al., 1996).

The use of specific PGR treatments to progress through the steps of SE reflects how phytohormone concentrations change in developing embryos. In this way, a particular SE step can be determined by measuring the endogenous levels of specific phytohormones. The examination of endogenous phytohormones during the process of SE in conifers has so far relied on the methods and instrumentation available in the time of the experiments. Fundamental information about changes in auxin, CK, and ABA concentrations in somatic embryos during SE were first published in the late 1990s (Find, 1997; Jourdain et al., 1997; Vagner et al., 1998, 1999b). These studies combined the HPLC method with ELISA/RIA for purification, fractionation, and quantification. However, this methodology only enabled researchers to measure the levels of fundamental phytohormones and some of their metabolites during SE. Jourdain et al. (1997) measured hormone concentrations in embryogenic cultures of larch and found high levels of 2,4-D, IAA-Asp, ABA-GE, and iPR, but low levels of BA, in early somatic embryos. Later von Aderkas et al. (2001) added to these results by reporting an increase in ABA and ABA-GE, as well as in IAA and IAA-Asp levels during the maturation of larch somatic embryos. They also revealed that zeatin (Z) and zeatin 9-riboside (ZR) levels fluctuate during the different phases of embryonic development and that there is a slight and transient increase in iP and iPR levels at the end of maturation. The profile of the analyzed phytohormones was comparable to what had been obtained for larch zygotic embryos. Other research has suggested that alterations in ABA levels are correlated with the quality of germination in spruce somatic embryos (Find, 1997).

Vagner et al. (1998) performed a detailed study of the phytohormonal changes that occur during SE in spruce with cell suspensions of Norway spruce embryos at proliferation and at the start of maturation. They later repeated the experiments by culturing Norway spruce embryos at the proliferation and maturation stages in agar medium (Vagner et al., 1999b). In these experiments, PEG was used to accelerate embryo maturation. The results showed that different stages of embryo development and maturation were correlated with distinct endogenous phytohormone profiles; there were dramatic changes in ABA, CK, and IAA levels, as well as in the production of ethylene.

Until now, the data regarding endogenous phytohormone levels in somatic embryos have been insufficient for the precise characterization of individual SE developmental steps in conifers. Research into the levels of certain phytohormones at different stages of SE can be further applied to investigating crosstalk between the signaling pathways that influence these regulators, as hormone action depends on concentration, a correct localization and interaction with specific receptors (Frebort et al., 2011). The coordinated interactions of phytohormone signaling pathways are considered to be the main mechanism regulating both plant and embryonic development. Nevertheless, the elucidation of how embryos, and then plants, develop on a physiological and biochemical level continues to require detailed endogenous hormone profiles for the distinct phases of development.

The main objective of our research was to provide a detailed analysis of endogenous phytohormones in the somatic embryos of Norway spruce during SE. We utilized advanced high performance liquid chromatography electrospray tandem-mass spectrometry (HPLC-ESI-MS/MS) to determine the profiles of a wide range of phytohormones throughout SE, i.e., from proliferation to germination. We included the main groups of phytohormones that have been studied in previous experiments, such as auxins, CKs, and ABA, along with their numerous metabolic forms, as well as other compounds that have not yet been investigated in relation to SE in conifers, e.g., jasmonates, SA, and BzA. The wide array of detected phytohormones and their fluctuating levels reveal how different phytohormone derivatives are involved in specific steps of Norway spruce SE, and the presented data could be used to elucidate the biochemical changes that occur during different steps of SE.

## Materials and Methods

### Cultivation of Plant Material

The embryogenic culture of *Picea abies* (L.) H. Karst, genotype AFO 541, was obtained from AFOCEL (AFOCEL, Nangis, France). During proliferation, the culture was grown on GD medium (Gupta and Durzan, 1986) solidified with 0.75% agar (Sigma-Aldrich, St. Louis, MO, United States), pH adjusted to 5.8 before autoclaving, and supplemented with 5 μM 2,4-D, 2 μM kinetin, 2 μM BA (all Sigma-Aldrich), and 30 g/L sucrose (Lachema, Brno, Czech Republic). All of the organic components, except sucrose, were separately prepared and diluted, filter-sterilized and added to the cooled, autoclaved media. The embryogenic culture was incubated in Magenta vessels (Sigma-Aldrich) containing 40 mL of fresh medium at 23 ± 1°C in the dark, and was subcultured weekly. Liquid GD medium, in which the CKs and auxin were substituted by 20 μM ABA (Sigma-Aldrich) and 3.75% PEG 4000 (Sigma-Aldrich), was used to promote the maturation of somatic embryos. The PEG solution was separately autoclaved. The ABA solution was filter-sterilized, after which both solutions were added to the medium after autoclaving. During maturation, cultures were subcultured weekly into fresh medium in Magenta vessels with membrane rafts (Osmotek, Rehovot, Israel) and incubated at 23 ± 1°C in the dark for 5 weeks. After the maturation stage, fully developed embryos were selected and desiccated by transferring them onto dry paper in small Petri dishes (3 cm diameter) that were kept open within large Petri dishes (18 cm diameter) that had several layers of paper wetted by sterile water to maintain high humidity. The large Petri dishes were covered with lids, sealed with parafilm, and then incubated in a cultivation room under a 12 h photoperiod at 18 ± 1°C for 3 weeks. The desiccated embryos were transferred into Magenta dishes filled with 40 mL of half-strength GD medium that was solidified by 0.75% agar, did not include any phytohormones, supplemented with sucrose (1%) and active charcoal (0.4%), and pH adjusted to 5.8 before autoclaving. The dishes containing embryos were placed in a cultivation room with a 12 h photoperiod at 23 ± 1°C for 1 week (Gemperlova et al., 2009).

**FIGURE 1:**
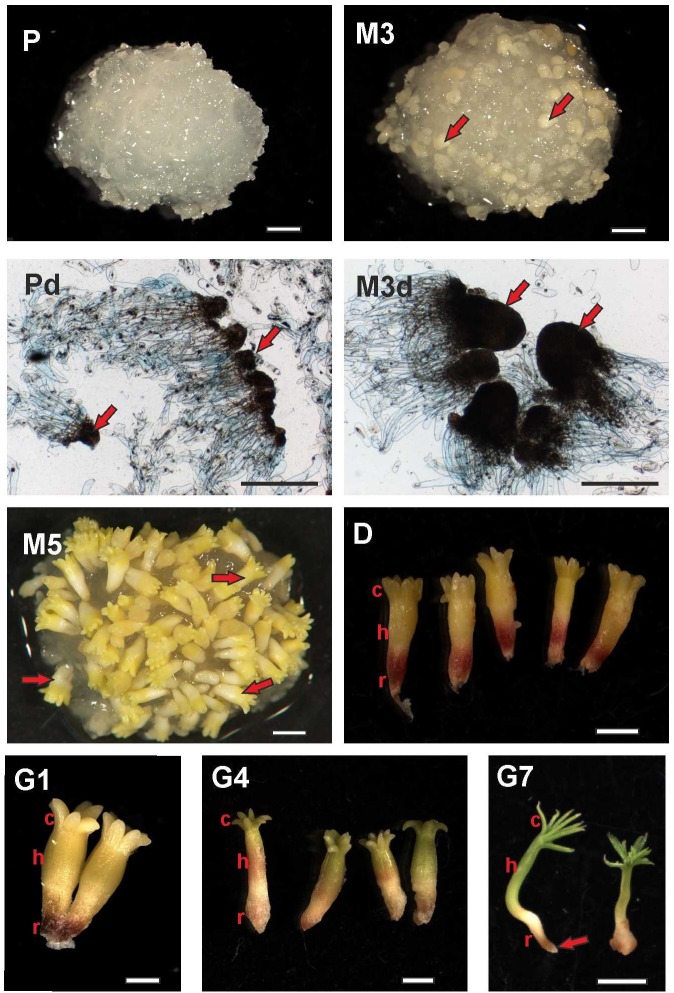
Documentation of *Picea abies* somatic embryos at distinct phases of somatic embryogenesis. The developmental steps shown here correspond to the time points when samples for phytohormone analyses were collected. P – Embryogenic suspensor mass during proliferation (scale bar represents 1 mm); Pd – Detail (microphotograph) of early somatic embryos during proliferation, with *arrows* showing embryonal heads (scale bar represents 500 μm); M3 – Three weeks after maturation, with *arrows* showing prolonged embryonal heads (scale bar represents 1 mm); M3d – Detail (microphotograph) of embryos during the 3rd week of maturation, with *arrows* showing prolonged embryonal heads (scale bar represents 500 μm); M5 – Mature somatic embryos after 5 weeks of maturation, with *arrows* showing mature somatic embryos (scale bar represents 1 mm); D – Single somatic embryos over 3 weeks of desiccation (c, cotyledons; h, hypocotyl; r, root tip; scale bar represents 1 mm); G1 – Embryos at the start of germination (24 h of germination; scale bar represents 1 mm); G4 – Embryos after 4 days of germination (scale bar represents 1 mm); G7 – Emblings after 1 week of germination (somatic embryos start their development as emblings; c, green growing cotyledons; h, prolonged hypocotyls; r, root tip; *arrow* points to protruding root; scale bar represents 5 mm).

The developmental stages are documented in **Figure 1**. All images were recorded using a Nikon DS-5M digital camera and processed using the Nis-Elements AR 3.0 (Laboratory Imaging, Prague, Czech Republic) computer image analysis system. Macrophotographs were taken using a SMZ 1500 stereomicroscope (Nikon, Tokyo, Japan). The microscopic preparations were examined using a Jenaval transmission light microscope (Zeiss, Oberkochen, Germany). The ESM material was placed on a microscopic slide and treated with one drop of 0.04% trypan blue (Sigma-Aldrich). A glass coverslip was then placed onto the ESM after 2 min of incubation, after which the dye was rinsed out with distilled water.

### Phytohormone Analysis

Phytohormone concentrations were measured at nine time points over the course of somatic embryo development, including proliferation (P) and maturation (M3, M5) (the samples were collected just before media was changed), 1, 2, and 3 weeks of desiccation (D1, D2, and D3) and 1, 4, and 7 days of germination (G1, G4, and G7). The details and abbreviations are provided in the Section “Results” and **Figure 1**. The plant samples were dried on cotton wool, then frozen in liquid nitrogen and stored at -80°C until analysis. At least five biological replicates (samples grown at the same time) were used for each analysis.

The analysis of plant hormones was carried out as described in Dobrev and Vankova (2012) and Djilianov et al. (2013). A ca. 100 mg fresh weight aliquot of frozen plant material was homogenized in liquid nitrogen by mortar and pestle. Cold extraction buffer (methanol/water/formic acid, 15/4/1, v/v/v, -20°C, 500 μL) was then added to the plant homogenates, along with a mixture of stable isotope-labeled internal standards (10 pmol). The following internal standards were used: [^13^C_6_]IAA (Cambridge Isotope Laboratories, Tewksbury, MA, United States); [^2^H_5_][^15^N_1_]IAA-Asp (OlchemIm, Olomouc, Czech Republic); [^2^H_4_]SA (Sigma-Aldrich); [^2^H_3_]PA (NRC-PBI, Saskatoon, Canada); [^2^H_5_]JA (C-D-N Isotopes Inc., Pointe-Claire, Canada); [^2^H_6_]ABA (NRC-PBI); [^2^H_5_]*trans*Z; [^2^H_5_]*trans*ZR; [^2^H_5_]*trans*Z-7-glucoside (*trans*Z7G); [^2^H_5_]*trans*Z-9-glucoside (*trans*Z9G); [^2^H_5_]*trans*ZOG; [^2^H_5_]*trans*ZROG; [^2^H_5_]*trans*ZRMP; [^2^H_3_]DHZ; [^2^H_3_]DHZR; [^2^H_3_]DHZ-9-glucoside (DHZ9G); [^2^H_6_]iP; [^2^H_6_]iPR; [^2^H_6_]iP-7-glucoside (iP7G); [^2^H_6_]iP-9-glucoside (iP9G); [^2^H_6_]iPRMP; and [^2^H_7_]BA (all CK standards were from OlchemIm, Olomouc, Czech Republic; the system of CK abbreviations adopted and modified according to Kaminek et al., 2000). For auxin metabolites with unavailable labeled equivalents, [^13^C_6_]IAA was used as an internal standard. The concentrations of *cis*Z derivatives were determined based on the retention times and mass spectra of unlabelled standards and the response ratio of their *trans*Z counterparts. All unlabelled standards were purchased from OlchemIm. Reversed-phase and ion-exchange chromatography (Oasis-MCX, Waters, Milford, MA, United States) resulted in two fractions: (1) fraction A (eluted with methanol), which contained hormones of acidic and neutral character (auxins, ABA, SA, JA, and their derivatives); and (2) fraction B (eluted with 0.35 M NH_4_OH in 70% methanol), which contained the hormones of basic character (CKs). Fractions were evaporated to dryness in a vacuum concentrator and dissolved in 30 μL of 10% methanol. An aliquot (10 μL) from each fraction was separately analyzed by HPLC with Ultimate 3000 (Dionex, Sunnyvale, CA, United States) that was coupled to a hybrid triple quadrupole/linear ion trap mass spectrometer (3200 Q TRAP, Applied Biosystems, Foster City, CA, United States) set in selected reaction-monitoring mode. The mass spectrometer was set at electrospray ionization mode, which was negative for fraction A and positive for fraction B. The ion source parameters were as follows: ion source voltage -4,000 V (negative mode) or +4,500 V (positive mode); nebulizer gas 50 psi; heater gas 60 psi; curtain gas 20 psi; heater gas temperature 500°C. The phytohormones were quantified using the isotope dilution method with multilevel calibration curves. All the gathered data were processed with Analyst 1.5 software (Applied Biosystems). The phytohormone concentrations were calculated as amount per 1 g of dry weight plant material because embryos have different water contents at different developmental stages (**Supplementary Table S1**).

### Statistical and Principal Components Analysis

The five independent biological replicates at each time point were analyzed with standard descriptive statistics and a Dixon test was used to exclude any outlying values. All source data are summarized in **Supplementary Table S2**.

Principal components analysis (PCA) was carried out summarizing correlations between the nine individual embryo developmental stages and the dynamics of the total concentrations of four particular phytohormone groups (auxins, CKs, ABA, and jasmonates) and two individual phytohormone species (SA and BzA). The first components of PCA (PC1 and PC2) were considered (**Supplementary Figure S1** and **Supplementary Table S3**). The Microcal Origin Pro 2018 statistical package was used.

## Results

### Documentation of *P. abies* Somatic Embryos at Selected Developmental Stages

Selected stages of SE were recorded when the embryogenic cultures were sampled for later phytohormone analyses (shown in **Figure 1**). The samples for proliferation (P) and the 3rd week after maturation (M3) stages were entirely within the ESM. The proliferation phase sample consisted of early somatic embryos and polyembryogenic complexes with small meristematic heads and long suspensors (**Figure 1**; P, Pd) while the sample from the 3rd week of maturation contained embryos with longer meristematic heads and shorter suspensors (**Figure 1**; M3, M3d). At the end of maturation (M5), the samples contained embryos that had separated from the remaining mass (**Figure 1**; M5). These fully developed, mature embryos were slight and long, consisting of apical and root meristem, root caps, hypocotyls, and a ring of cotyledons around the apical meristem. At the start of desiccation, the embryos grew slowly, the cotyledons became green and the root pool of embryos was red. No morphological changes were discerned during the 2nd and 3rd weeks of desiccation (**Figure 1**; D). The embryos enlarged soon after being transferred to germinating medium, with embryos one day after germination shown in **Figure 1** (G1). The hypocotyls became prolonged and green cotyledons began to grow on the 4th day after germination (Fig 1; G4). Emblings developed 1 week after germination, indicating that shoot development was continuing and root formation could start (**Figure 1**; G7).

### Hormonal Profiling

**FIGURE 2:**
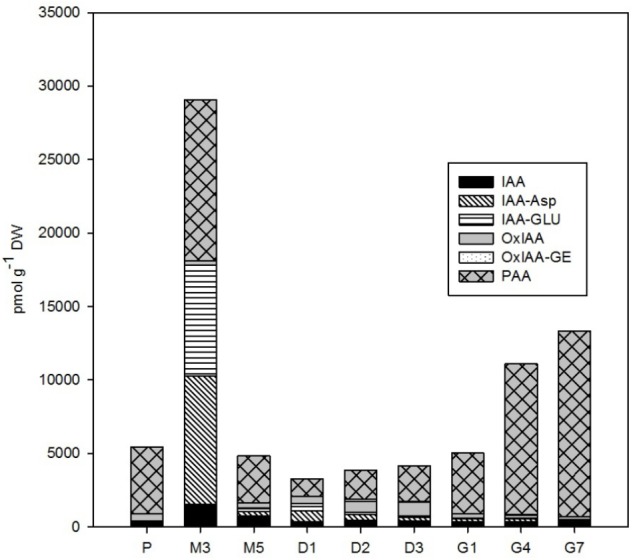
The concentrations of auxins in the *Picea abies* embryogenic cultures at various phases of somatic embryogenesis. IAA, indole-3-acetic acid; IAA-Asp, IAA-aspartate; IAA-GLU, IAA-glutamate; OxIAA, oxo-IAA; OxIAA-GE, oxo-IAA-glucosylester; PAA, phenylacetic acid. x-axis: P – proliferation, M3 – 3 weeks of maturation, M5 – 5 weeks of maturation, D1 – 1 week of desiccation, D2 – 2 weeks of desiccation, D3 – 3 weeks of desiccation, G1 – 1 day of germination, G4 – 4 days of germination, G7 – 7 days of germination.

**FIGURE 3:**
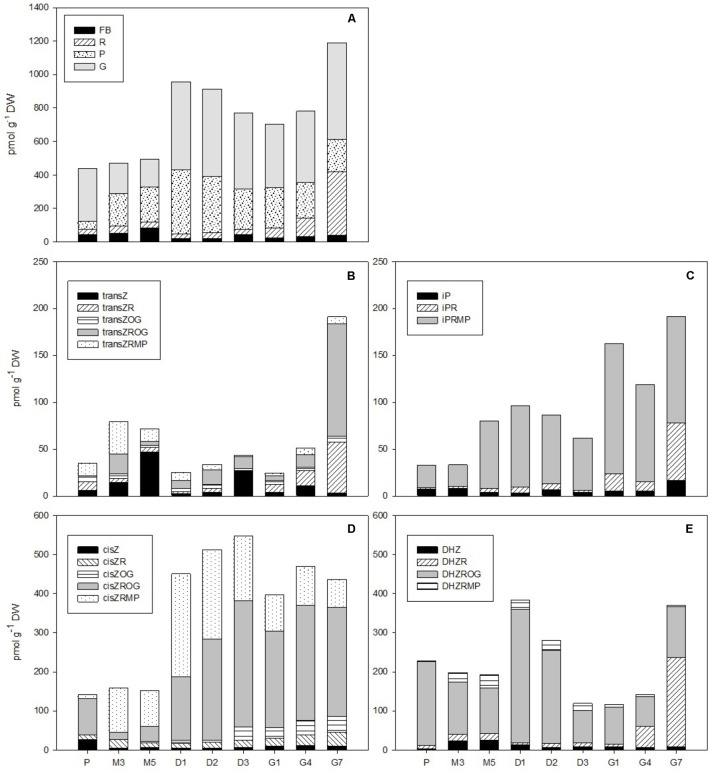
The concentrations of cytokinins in the *Picea abies* embryogenic cultures at various phases of somatic embryogenesis. **(A)** Free bases (FB), ribosides (R), phosphates (P), and *O*-glucosides (G). **(B)** *Trans*-zeatin types: *trans*Z, *trans*-zeatin; *trans*ZR, *trans*-zeatin 9-riboside; *trans*ZOG, *trans*-zeatin *O*-glucoside; *trans*ZROG, *trans*-zeatin 9-riboside *O*-glucoside; *trans*ZRMP, *trans*-zeatin 9-riboside-5′-monophosphate. **(C)** *N^6^*-(Δ^2^-isopentenyl)adenine types: iP, *N^6^*-(Δ^2^-isopentenyl)adenine; iPR, *N^6^*-(Δ^2^-isopentenyl)adenosine; iPRMP, *N^6^*-(Δ^2^-isopentenyl)adenosine-5′-monophosphate. **(D)** *cis*-zeatin types: *cis*Z, *cis*-zeatin; *cis*ZR, *cis*-zeatin 9-riboside; *cis*ZOG, *cis*-zeatin *O*-glucoside; *cis*ZROG, *cis*-zeatin 9-riboside *O*-glucoside; *cis*ZRMP, *cis*-zeatin 9-riboside-5′-monophosphate. **(E)** Dihydrozeatin types: DHZ, dihydrozeatin; DHZR, dihydrozeatin 9-riboside; DHZROG, dihydrozeatin 9-riboside *O*-glucoside; DHZRMP, dihydrozeatin 9-riboside-5′-monophosphate. x-axis: The details are as described in **Figure 2**.

**FIGURE 4:**
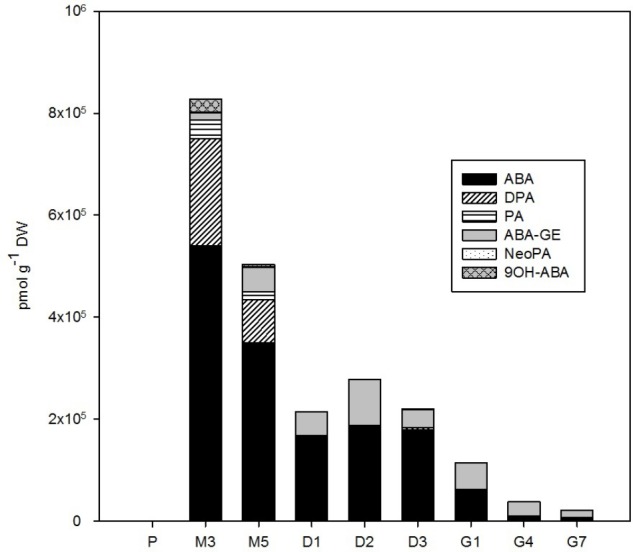
The concentration of ABA and its derivatives in the *Picea abies* embryogenic cultures at various phases of somatic embryogenesis. ABA, abscisic acid; ABA-GE, ABA-glucosylester; PA, phaseic acid; DPA, dihydrophaseic acid; NeoPA, neophaseic acid; 9OH-ABA, 9-hydroxy-ABA. x-axis: The details are as described in **Figure 2**.

**FIGURE 5:**
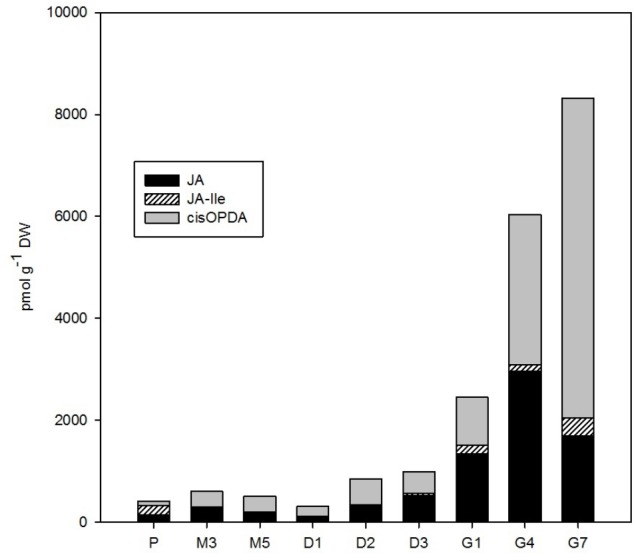
The concentrations of jasmonates in the *Picea abies* embryogenic cultures at various phases of somatic embryogenesis. JA, jasmonic acid; JA-Ile, JA-isoleucine; *cis*OPDA, *cis*-(+)-12-oxo-phytodienoic acid. x-axis: The details are as described in **Figure 2**.

**FIGURE 6:**
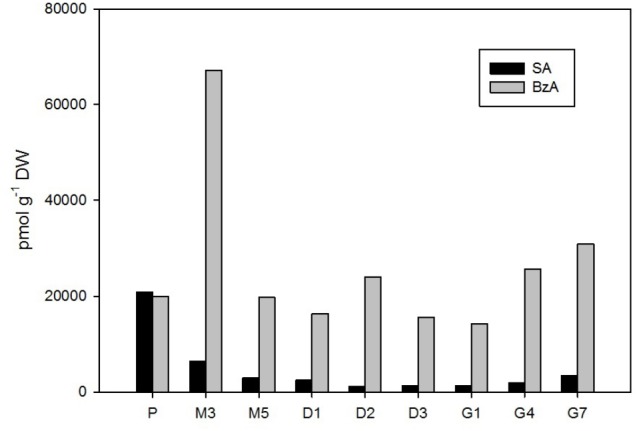
The concentrations of salicylic and benzoic acid in the *Picea abies* embryogenic cultures at various phases of somatic embryogenesis. SA, salicylic acid; BzA, benzoic acid. x-axis: The details are as described in **Figure 2**.

The phytohormone analysis was performed following dual-mode solid-phase extraction through HPLC-electrospray tandem-mass spectrometry, which enabled simultaneous and highly reliable identification and quantification of about 35 phytohormones including auxins, CKs, ABA, jasmonates, SA, and their conjugates. The concentrations of these distinct phytohormones varied considerably over the course of SE, as is demonstrated in **Figures 2**–**6** and **Supplementary Table S2**.

### Auxins

A wide spectrum of endogenous auxins, including both indole and non-indole analogs, was detected during the *P. abies* SE process (**Figure 2**). PAA, a non-indole phenolic compound with weak auxin activity, was the most abundant auxin with concentrations ranging from 1,180 to 12,650 pmol/g DW during SE. Among the indole auxins, free IAA and its amino acid conjugates IAA-Asp and IAA-GLU predominated. PAA and the indole auxins were very prominent especially during the start of maturation, reaching a maximum at the 3rd week of maturation (M3). The concentrations of all of these compounds then sharply decreased and stayed low during desiccation, whereas a mild presence of oxIAA, the major IAA catabolite, was noted at the end of desiccation (D3). The next stage of SE, germination, was associated with a sharp increase in PAA content, which reached over 12,500 pmol/g DW after 7 days (G7), but low concentrations of the indole auxins, with levels below 900 pmol/g DW, similar to what had been observed during proliferation (P) (**Figure 2**). It should also be noted that there were exceptionally high amounts of 2,4-D (up to 380,000 pmol/g DW) in the embryogenic culture at proliferation (P), which was due to the presence of this synthetic auxin in the proliferation medium, as also indicated by its considerably decreased levels (by almost three orders of magnitude) in embryos during the subsequent stages of SE (data not shown).

### Cytokinins

In total, 17 isoprenoid CKs were detected over the course of *P. abies* SE. This included bioactive forms (free bases), transport forms (ribosides), storage forms (*O-*glucosides), and CK phosphates that represent the immediate biosynthetic precursors. Interestingly, no CK *N-*glucosides (deactivation forms of CKs) were found during the development of somatic embryos. The total CK concentration varied considerably during different SE stages, ranging from hundreds to over a thousand of picomoles per gram DW and reaching maxima at the start of desiccation (D1) and at the end of germination (G7) (**Figure 3A**). The first maximum, which was observed at the beginning of desiccation (ca. 960 pmol/g DW at D1), was caused by *O-*glucoside and CK phosphate peaks, whereas the second maximum, which was noted during germination (ca. 1,200 pmol/g DW at G7), can be explained by an increase in bioactive and particularly transport CK levels.

The total CK pool in somatic embryos was composed of *trans*Z, *cis*Z (5 forms each), DHZ (4 forms), and iP (3 forms) derivatives. The *trans*Z-, *cis*Z-, DHZ-, and iP-type CK profiles were characterized separately (**Figures 3B–E**). In general, the *cis*Z- and DHZ-types predominated throughout the entire SE process and exhibited maxima at desiccation (ca. 550 and 390 pmol/g DW, respectively) and germination (ca. 470 and 370 pmol/g DW, respectively) (**Figures 3D,E**). The *trans*Z- and iP-types were less abundant in embryos, occurring at moderate concentrations (below 100 pmol/g DW) throughout SE and increasing toward the end of germination (G7) to concentrations of up to 190 pmol/g DW (**Figures 3B,C**).

Five forms of *cis*Z-type CKs were found in embryos during *P. abies* SE (**Figure 3D**). Among them, *cis*ZROG, with two maxima at desiccation and germination (ca. 325 and 295 pmol/g DW at D3 and G4, respectively), and *cis*ZRMP, which peaked at the beginning of desiccation (ca. 265 pmol/g DW at D1), were the most plentiful. Other forms, namely, *cis*Z, *cis*ZR, and *cis*ZOG, were less abundant, reaching concentrations of around 40 pmol/g DW at their respective maxima. The transition from desiccation to germination was associated with a slight increase in *cis*Z and *cis*ZR levels and a decrease in *cis*ZOG and *cis*ZROG levels (**Figure 3D**).

The content of DHZ-type CKs in *P. abies* embryos during SE reached a maximum at the start of desiccation, and this was largely due to a distinct DHZROG (up to 345 pmol/g DW at D1) peak (**Figure 3E**). DHZROG was the most abundant DHZ-type CK throughout SE. The concentration of DHZR grew strongly in germinating embryos (230 pmol/g DW at G7) and resulted in another maximum at the end of germination. Two other derivatives, DHZ and DHZRMP, were also detected, but they occurred in amounts that did not exceed 35 pmol/g DW throughout SE (**Figure 3E**).

The *trans*Z-type CKs were detected at rather low concentrations during *P. abies* SE (**Figure 3B**). The amount of *trans*Z-type CK slightly grew during maturation, and this increase was particularly associated with increasing concentrations of *trans*Z and *trans*ZRMP. A more pronounced rise in *trans*Z-type CK levels was seen at germination, and can be mainly attributed by increased concentrations of *trans*ZR and especially *trans*ZROG. The other detected *trans*Z-type CKs fluctuated throughout the SE process with maxima that did not exceed tens of picomols/g DW (**Figure 3B**).

Among the iP-type CKs, iPRMP represented the most abundant form found in *P. abies* embryos during SE (**Figure 3C**). The iPRMP concentration increased slightly throughout SE and exhibited two peaks: the first at the beginning of desiccation (87 pmol/g DW at D1) and another peak at the start of germination (139 pmol/g DW at G1). The levels of the two other iP derivatives, iP and iPR, were rather low (not exceeding 10 pmol/g DW) during proliferation, maturation, and desiccation, but substantially increased during germination (up to 17 and 61 pmol/g DW, respectively) (**Figure 3C**).

Proliferation was characterized by extremely high levels of BA (ranging from 26,000 to 51,000 pmol/g DW), which reflected the presence of aromatic CKs (BA and kinetin) in the medium. As development progressed, the BA concentration in embryos decreased 100- to 1,000-fold. No other BA derivatives were detected throughout the course of SE; this indicates that BA taken up by the embryogenic culture is metabolically stable (data not shown).

### Abscisic Acid

The amount of endogenous ABA and its derivatives in embryos during *P. abies* SE (**Figure 4**) strongly depended on the presence of exogenously supplied ABA in the cultivation medium. During proliferation, when the cultures were grown on ABA-free medium, the endogenous concentration of ABA in embryos was 142 pmol/g DW. Other ABA derivatives were also detected at proliferation; the catabolites PA and DPA occurred at concentrations below 20 pmol/g DW and ABA-GE was found at a concentration of 131 pmol/g DW. Maturation was induced by the exogenous application of ABA to the medium, which resulted in a tremendous increase (almost 4,000-fold) in embryogenic culture ABA levels from proliferation to maturation. During maturation, when embryogenic cultures were kept on media supplemented with ABA, the ABA concentration, as well as the concentrations of its deactivation products 9OH-ABA, PA and DPA, in embryos decreased substantially and continuously until the start of desiccation. The ABA concentration remained more or less constant during desiccation and declined further during germination. The compounds 9OH-ABA, PA, and DPA were no longer detected following the start of desiccation. However, another ABA metabolite, ABA-GE, was detected at moderate concentrations throughout the whole SE process, and had a maximum at desiccation (89,000 pmol/g DW at D3; **Figure 4**).

### Jasmonates

The total content of jasmonates in *P. abies* embryos gradually increased over the course of SE from ca. 400–1,000 pmol/g DW at proliferation, maturation, and desiccation up to 8,300 pmol/g DW at the end of germination (G7; **Figure 5**). JA and its precursor *cis*OPDA were the most prominent jasmonates detected during SE and exhibited similar dynamics until the start of germination. During germination, the concentration of *cis*OPDA continued to grow progressively (up to 6,300 pmol/g DW at G7), whereas JA content sharply decreased after the 4th day of germination (to 1,700 pmol/g DW at G7). The supposedly active form of JA, JA-Ile, was less abundant during SE, reaching a maximum of 360 pmol/g DW (at G7; **Figure 5**).

### Salicylic and Benzoic Acids

Two phenolic compounds, SA and BzA, were found at relatively high concentrations in *P. abies* embryos during the entire SE process (**Figure 6**). The maximal content of SA (21,000 pmol/g DW) was found in early embryos during the proliferation stage. SA levels then considerably declined during maturation and desiccation (to 1,200 pmol/g DW at D2 and D3), but a slight increase was recorded in germinated embryos (up to 3,400 pmol/g DW at G7).

The concentration of BzA substantially exceeded that of SA throughout SE (except for proliferation), and maximal BzA levels in embryos were noted during the 3rd week of maturation (67,000 pmol/g DW at M3). Two smaller peaks were observed at desiccation (24,000 pmol/g DW at D2) and at the end of germination (31,000 pmol/g DW at G7; **Figure 6**). Interestingly, BzA had similar dynamics as another phenolic acid PAA, which was, nevertheless, found at far lower concentrations; this compound is, however, categorized as a non-indole auxin in this paper and the corresponding data are presented above (**Figure 2**).

### Principal Components Analysis

Three and four principal components account for 94.6 and 99.1% of the cumulative variance, respectively. Thus four principal components were used for the analysis. The correlation matrix is presented in the **Supplementary Table S3**. There is a strong collinearity between auxins and BzA (*r* = 0.97) across the embryo developmental stages. Generally, there is a significant correlation between the phytohormone concentrations with exception of SA and BzA, SA and auxins, and jasmonates and BzA. In the **Supplementary Figure S1**, the first two components (PC1 and PC2) of PC space of the embryo developmental stages are demonstrated. The first two components accounted for 77% of the variance observed. PC1 strongly correlates with auxins, ABA and BzA, and PC2 with jasmonates and CKs (see Factor loadings in **Supplementary Table S3**).

## Discussion

A number of the phytohormone groups analyzed in our experiments were present in embryos throughout the entire SE process. We characterized the profiles and dynamics of individual phytohormone forms to postulate how they might be involved in regulating embryonic development at particular steps of SE.

### Proliferation

Proliferation is the active step of SE going on a medium supplemented with auxin (2,4-D) and aromatic CKs (BA and kinetin). During proliferation, the ESM is composed of early somatic embryos and polyembryogenic complexes (Svobodova et al., 1999; Gemperlova et al., 2009), i.e., two kinds of cells, meristematic and suspensor, exist (**Figure 1**). As the ESM develops, meristems enlarge and suspensors prolong while older structures are simultaneously eliminated as a prerequisite for the formation of new embryos and complexes. Thus, the ESM always consists of embryogenic structures at distinct stages of development. The endogenous phytohormone profile is a proportional representation of individual ESM components and also shows the concentrations of various exogenous PGRs that are necessary for successful proliferation.

We found very high concentrations of 2,4-D (up to 380,000 pmol/g DW) in the *P. abies* EMS during proliferation (data not shown). The 2,4-D is an auxin herbicide (Song, 2014) that is known to be necessary for the optimal proliferation of numerous embryogenic cultures of conifers (Vondrakova et al., 2016). It is more stable than natural auxins in plants mediating auxin responses during their growth and/or senescence (Grossmann, 2010). The concentrations of endogenous indole auxins were rather low in early embryos during proliferation (ca. 900 pmol/g DW in total), with IAA and its primary catabolite oxIAA as the most prevalent forms (**Figure 2**). Surprisingly, a non-indole phenolic compound with weak auxin activity, PAA, was found at much higher concentration (4,530 pmol/g DW) in spruce ESM than indole auxins (**Figure 2**). The role of indole auxins during proliferation of conifer embryogenic cultures seems to be rather ambiguous, as both inhibitory and stimulatory effects have been recorded (Norgaard and Krogstrup, 1991; Liao et al., 2008; Vondrakova et al., 2011). In contrast, PAA involvement in conifer embryogenesis has not previously been reported. PAA may have a distinct role from IAA in SE as PAA transport and biosynthesis have been suggested to proceed independently of IAA (Sauer et al., 2013; Sugawara et al., 2015).

Cytokinins play essential roles in both the induction and proliferation of embryogenic cultures, with aromatic CKs as integral constituents of media used during the induction and proliferation stages in conifer SE (Norgaard and Krogstrup, 1991; Jain et al., 1995; Guevin and Kirby, 1997). Although the HPLC-ESI-MS/MS method also allowed us to identify aromatic BA-type CKs, the emphasis was nevertheless on measuring endogenous isoprenoid CK levels. The maximum BA concentration was observed at the proliferation stage (data not shown), and reflects the presence of aromatic CKs (BA and kinetin) in the medium. The absence of other BA derivatives, for example, ribosides and *N*-glucosides, indicated substantial BA metabolic stability within the culture. Regarding isoprenoid CKs, 17 forms, including bioactive, transport and storage forms as well as CK phosphates, were detected at proliferation. The total isoprenoid CK content at this stage was surprisingly low, not exceeding 450 pmol/g DW (**Figure 3**). Interestingly, no deactivation forms (*N-*glucosides) of isoprenoid CKs were found, which, together with the aforementioned absence of BA-*N*-glucosides, suggests that the CK-*N-*glucosyltransferase pathway, being relatively common within the plant kingdom, e.g., (Vankova, 1999; Wang et al., 2011, 2013; Jiskrova et al., 2016; Smehilova et al., 2016) and playing a role in e.g., the growth of conifer buds (Zhang et al., 2003) and their organogenesis *in vitro* (Montalbán et al., 2013), is missing or insignificant in the SE process. The *cis*Z- and DHZ-types represented the major endogenous CKs present in the ESM. The prevailing CK derivatives, *cis*ZROG and DHZROG, could be interpreted as the principal storage CK forms at the proliferation stage of *P. abies* embryos.

Relatively low amounts of ABA and its derivatives were found in *P. abies* ESM at proliferation (**Figure 4**), and this seems to be common for the initial phases of embryonic development in other coniferous genera as well (Stasolla and Yeung, 2003). Similarly, we found rather low endogenous levels of jasmonates in proliferating embryos (**Figure 5**). While endogenous ABA may be involved in mediating the multiplication and destruction of early embryos during proliferation, a function for jasmonates in the early developmental stages of conifer embryogenic cultures has not yet been described. In contrast, SA and BzA were present at relatively high concentrations in the proliferating ESM (**Figure 6**). Despite these high levels, the role of these phenolic compounds during proliferation remains unclear and has yet to be elucidated. SA has been reported to participate in plant mechanisms that defend against microbial infections (An and Mou, 2011) and, in this way, could influence the immunity of proliferating embryos.

To summarize, dramatic developmental processes occur in embryos during proliferation. Although this step of SE was characterized by low endogenous levels of most phytohormones (except for SA and BzA), intense hormonal regulation of the multiplication/destruction processes of embryogenic structures is postulated.

### Maturation

An embryogenic culture can be triggered to transition from early to mature embryos by replacing the 2,4-D and CKs in the growth medium with ABA. High exogenous ABA concentrations (20 μM) are needed throughout the maturation process; the absence or low amounts of ABA in the medium may lead to aberrant development, characterized by numerous malformed embryos (Fischerova et al., 2008). The *P. abies* embryogenic structures at maturation are shown in **Figure 1**. Hormonal analyses were performed twice during maturation: 3 weeks after start of maturation (M3), when embryo polarization was occurring; and 5 weeks after start of maturation (M5), when mature embryos had separated from the remaining mass (**Figure 1**).

Maturation is initiated by exogenous application of ABA, so, as expected, a sharp increase in the endogenous levels of ABA and its derivatives in the ESM was observed following passage to maturation medium, reaching a peak at M3 (**Figure 4**). This time point represents inhibition of the proliferation process as enhanced endogenous ABA concentrations at maturation seem to suppress the formation of new early embryos and embryogenic complexes and regulate the further development of certain embryos into mature forms (Filonova et al., 2000; Schwarzerova et al., 2010). The concentration maxima for most auxins – both indole forms (IAA and its amino acid conjugates) and the non-indole PAA – also occurred at time point M3 (**Figure 2**). This finding agrees with previous research, which found a marked increase in the endogenous levels of ABA, ABA-GE, IAA, and IAA-Asp during maturation of larch somatic embryos (von Aderkas et al., 2001) and high concentrations of the IAA and IAA-Asp in zygotic Douglas fir embryos (Chiwocha and von Aderkas, 2002). It seems likely that the timing of these maxima correlates with the polarization of somatic embryos (Vagner et al., 1999a,b).

Contrary to auxins, CKs were detected at rather low concentrations in maturing *P. abies* embryos, corresponding with their reduced levels at proliferation. The relationship between auxin and CK contents is evidently decisive for embryonic development (Su et al., 2011), as the high auxin/CK ratio at maturation seems to be a prerequisite for proper formation of apical and root meristems, optimal development of root caps, hypocotyls and cotyledons, and differentiation of basic endogenous structures, including tracheids (Svobodova et al., 1999). However, the molecular mechanisms underlying auxin–CK interactions remain largely unknown.

There were relatively low endogenous levels of jasmonates in the maturating ESM (**Figure 5**). The same holds for the concentration of SA, which progressively decreased in embryos during maturation. On the other hand, the levels of another phenolic compound, BzA, peaked after 3 weeks of maturation (**Figure 6**) and then abruptly declined, at M5 reaching the same level that was observed during proliferation and exhibiting analogous dynamics as another phenolic compound with a weak auxin activity PAA (**Figure 2**).

In summary, a significant increase in the endogenous concentrations of auxins, ABA and BzA was recorded after 3 weeks of maturation. The 3rd week of maturation represents a pivotal moment in the development of somatic embryos and is characterized by embryo polarization and the formation of cotyledonary embryos. The established developmental changes are apparently regulated by coordinated crosstalk between individual phytohormone groups involved in metabolic and signaling pathways, a phenomenon which has also been proposed by other authors (e.g., Lorenzo and Solano, 2005; Sun et al., 2009; Linkies and Leubner-Metzger, 2012).

### Desiccation

During this step, which is often incorrectly designated a SE resting step, the mature somatic embryos are kept without any medium and thus, do not receive exogenous phytohormones. During the first part of desiccation, the spruce embryos grow slowly and their water content decreases (to ca. 20% DW); at the next stage, no noticeable morphological changes or water losses occur (Vondrakova, unpublished data).

The auxin/CK ratio in *P. abies* embryos was very low at desiccation. The indole auxin levels were minimal and did not vary considerably, whereas the concentration of PAA was markedly higher at the beginning of desiccation and started to grow during the next time points (**Figure 2**), suggesting that this non-indole auxin exerts a favorable effect on subsequent embryo germination. Although the auxin activity of PAA is substantially lower than that of indole auxins (Schneider et al., 1985), its mechanism of action is different and its endogenous level exceeded that of indole auxins in *P. abies* embryos (Simon and Petrasek, 2011; Sugawara et al., 2015). One of the CK concentration maxima occurred at the start of desiccation (**Figure 3**). This peak was caused by a steep increase in the levels of CK phosphates (particularly *cis*ZRMP) and *O-*glucosides (in particular *cis*ZROG and DHZROG), which remained high throughout the desiccation process. This finding indicates that storage CKs may accumulate in embryos during desiccation. The start of desiccation phase also included a sharp decline in endogenous ABA levels, as well as those of its derivatives (especially PA, DPA, and 9OH-ABA) (**Figure 4**). The decrease in endogenous ABA concentrations at the start and its relatively steady levels during desiccation seem to create conditions for successful germination, as has been previously demonstrated for other *Picea* species (Roberts et al., 1990; Find, 1997). Concentrations of the ABA storage conjugate ABA-GE, on the other hand, increased during desiccation. Therefore, it is possible that the increased “pool” of CK and ABA storage derivatives partially compensates for the loss of bioactive CK and ABA forms during this step.

A slight increase in the levels of both major jasmonates, JA and *cis*OPDA, was observed in desiccated embryos (**Figure 5**), and this change seems to precede optimal germination. The phenolic compounds SA and BzA were at their lowest levels during desiccation (except for a mild enhancement of BzA at D2) (**Figure 6**). These substances have been shown to regulate stress responses (Senaratna et al., 2003); in this way, their reduced concentrations during desiccation suggest that desiccated spruce embryos are not under stressful conditions.

To sum up, no distinct changes in phytohormone levels were found in *P. abies* embryos during the desiccation step of SE. However, desiccation did involve the formation of a “reserve pool” of storage CK and ABA derivatives, which may represent a strategy for balancing the reduction in bioactive CK and ABA forms that occurs during this developmental step.

### Germination

Embryos germinate on charcoal-containing media that is devoid of phytohormones and contains reduced nutrient and sucrose levels. After being transferred onto this media, the desiccated embryos absorb water, which is a prerequisite for their subsequent rapid growth and the active development that can begin as early as 1 week into germination. We performed the first germination phytohormone measurements at this time point (**Figure 1**).

There was a stark difference between the levels of indole and non-indole auxins during *P. abies* embryo germination. Whereas indole auxin concentrations remained steady and low during the course of germination, the sharp and progressive increase in PAA levels observed in germinating embryos since the first day continued throughout the whole week (**Figure 2**). The increasing levels of PAA observed during germination indicated that this non-indole auxin, unlike indole derivatives, is involved in the intense growth changes associated with embling development and the subsequent formation of cotyledons and roots. Sugawara et al. (2015) reported that PAA is prevalent throughout the plant kingdom and has an important role as an auxin in many aspects of plant growth and development. Although we identified overlapping regulatory roles for PAA and IAA, the distinct biosynthetic, transport and degradation pathways of non-indole and indole auxins are obvious and may underlie their contrasting concentration changes during Norway spruce embryo germination.

A noticeable increase in total CK content, which exhibited a similar trend as PAA, was found in *P. abies* embryos within the 1st week of germination (**Figure 3**). The ribosides (DHZR, *trans*ZR, and iPR) and *O*-glucosides (*trans*ZR*O*G) were mainly responsible for this change, while concentrations of bioactive free bases, except for iP, did not fluctuate much in germinating embryos. The level of CK phosphates, which represent primary products of CK biosynthesis (Sakakibara, 2006), slightly declined during germination; this finding might be due to the gradual enzymatic turnover of CK phosphates into the corresponding ribosides and/or free bases that are important for the formation and subsequent growth of the emblings. This view is supported by the fact that iP-, iPR-, and *trans*Z-type CKs, which are the first products of iPRMP conversion in developing plants (Sakakibara, 2006), were not present at high concentrations during earlier SE stages, but rather showed maximum levels at the end of germination.

The intense drop in the levels of endogenous ABA and its derivatives (mainly ABA-GE) observed in *P. abies* embryos at the start, and during the course, of germination (**Figure 4**) seems to be necessary to optimal progression of the germination process. This theory is supported by evidence that embryonic ABA is key to the induction and maintenance of seed dormancy, and also inhibits the transition from embryonic to germination growth (Rodriguez-Gacio et al., 2009).

Jasmonate levels also markedly increased during germination. A considerable increase in the endogenous levels of JA and its precursor, *cis*OPDA, was recorded in germinating embryos until the 4th day after the start of germination (**Figure 5**). Similarly, SA, and especially BzA, concentrations grew during germination, which suggests that they are involved in embling development even though these compounds have been shown to exert inhibitory effects in some plant species, for example, those of the genera Alliaria (Ribeiro et al., 2015). Yasin and Andreasen (2015) reported that increasing JA and SA concentrations are associated with the development of the vegetative axis in macaw palm seedlings.

To summarize, germination and the start of embling development in *P. abies* embryos involves a sequence of complex temporally and spatially balanced events associated with increased levels of the non-indole auxin PAA, total CKs and jasmonates, and decreased levels of ABA and its derivatives. This complex developmental stage is evidently influenced by both crosstalk between different phytohormones (Gomez-Cadenas et al., 2015) as well as crosstalk between hormones and the environment, e.g., light (Zdarska et al., 2015), with correct synchronization maintaining hormonal homeostasis during the germination process.

## Conclusion

Somatic embryogenesis in conifers is driven by a complex network of hormonal, metabolic and signaling pathways that respond to the strict regulation of particular developmental steps, from early somatic embryos to emblings, by exogenous PGR treatments. By taking advantage of advanced HPLC-ESI-MS/MS, we were able to identify more than 30 endogenous phytohormones including auxins, CKs, ABA and its derivatives, jasmonates and two phenolic compounds (SA and BzA), and follow their concentration changes during SE in Norway spruce. The PCA revealed especially an important role for auxins, ABA and BzA in maturated somatic embryos and a strong collinearity between auxins and BzA. According to our knowledge, we have provided evidence for the involvement of certain phytohormone derivatives, such as the non-indole auxin PAA, *cis*Z- and DHZ-type CKs, jasmonates and SA, in the development of conifer somatic embryos for the first time. This work represents the currently most comprehensive overview of plant hormones that are involved in SE and their concentration profiles over the proliferation, maturation, desiccation, and germination of conifer somatic embryos.

## Author Contributions

ZV, MV, and VM conceived and designed the research. ZV, PD, LF, and VM performed the experiments. ZV, PD, BP, and VM analyzed the data. ZV and VM wrote the manuscript. All authors have read and approved the final manuscript.

## Conflict of Interest Statement

The authors declare that the research was conducted in the absence of any commercial or financial relationships that could be construed as a potential conflict of interest.

## Supplementary Material

TABLE S1 Changes of dry weight (DW, %) in the *P. abies* embryogenic cultures during the process of somatic embryogenesis.

TABLE S2 Complete list of the distribution and endogenous levels (pmol/g DW) of phytohormones and their derivatives in the *P. abies* embryogenic cultures during the process of somatic embryogenesis.

TABLE S3 Principal components analysis (PCA) summarizing correlations between individual embryo developmental stages and the dynamics of the total concentrations of four particular phytohormone groups (auxins, CKs, ABA, and jasmonates) and two individual phytohormone species (salicylic and benzoic acids) in the *P. abies* embryogenic cultures.

FIGURE S1 Principal component analysis (PCA) space of the phytohormone concentrations examined, defined by the first two components (PC1 and PC2) for all embryo developmental stages in the *P. abies* embryogenic cultures. P, proliferation; M, maturation (M3, M5 – 3 and 5 weeks of maturation, respectively); D, desiccation (D1, D2, D3 – 1, 2, and 3 weeks of desiccation, respectively); G, germination (G1, G4, G7 – 1, 4, and 7 days of germination, respectively).

## Abbreviations

ABA: abscisic acid
ABA-GE: ABA-glucosylester
BA: *N*^6^-benzyladenine
BzA: benzoic acid
*cis*OPDA: *cis*-(+)-12-oxo-phytodienoic acid
*cis*Z: *cis*-zeatin
*cis*ZOG: *cis*-zeatin *O*-glucoside
*cis*ZR: *cis*-zeatin 9-riboside
*cis*ZRMP: *cis*-zeatin 9-riboside-5′-monophosphate
*cis*ZROG: *cis*-zeatin 9-riboside *O*-glucoside
CKs: cytokinins
2,4-D: 2,4 dichlorophenoxyacetic acid
DHZ: dihydrozeatin
DHZR: dihydrozeatin 9-riboside
DHZRMP: dihydrozeatin 9-riboside-5′-monophosphate
DHZROG: dihydrozeatin 9-riboside *O*-glucoside
DPA: dihydrophaseic acid
ESM: embryogenic suspensor mass
IAA: indole-3-acetic acid
IAA-Asp: IAA-aspartate
IAA-GLU: IAA-glutamate
iP: *N^6^*-(Δ^2^-isopentenyl)adenine
iPR: *N^6^*-(Δ^2^-isopentenyl)adenosine
iPRMP: *N^6^*-(Δ^2^-isopentenyl)adenosine-5′-monophosphate
JA: jasmonic acid
JA-Ile: JA-isoleucine
NeoPA: neophaseic acid
9OH-ABA: 9-hydroxy-ABA
OxIAA: oxo-IAA
OxIAA-GE: oxo-IAA-glucosylester
PA: phaseic acid
PAA: phenylacetic acid
PEG: polyethylene glycol
PGR: plant growth regulator
SA: salicylic acid
SE: somatic embryogenesis
*trans*Z: *trans*-zeatin
*trans*ZOG: *trans*-zeatin *O*-glucoside
*trans*ZR: *trans*-zeatin 9-riboside
*trans*ZRMP: *trans*-zeatin 9-riboside-5′-monophosphate
*trans*ZROG: *trans*-zeatin 9-riboside *O*-glucoside
