# Supplementary material for: Profiles of Endogenous Phytohormones Over the Course of Norway Spruce Somatic Embryogenesis
Source: Front Plant Sci. 2018 Sep 6;9:1283. doi: 10.3389/fpls.2018.01283 (PMC6136392; doi:10.3389/fpls.2018.01283)
Supplement: TABLE S1 — Changes of dry weight (DW, %) in the P. abies embryogenic cultures during the process of somatic embryogenesis. [file Table_1.DOCX]

**Table S1. Changes of dry weight (DW, %) in the *Picea abies* embryogenic cultures during the process of somatic embryogenesis.**

**(Data used for recalculating phytotohormone contents in fresh/dry weight.)**

Mean values (A) of three independent measurements are shown, including standard deviations (stds) and relative standard deviations (rstds).

|  | **P** | **M3** | **M5** | **D1** | **D2** | **D3** | **G1** | **G4** | **G7** |
| --- | --- | --- | --- | --- | --- | --- | --- | --- | --- |
| A | 6,50 | 9,40 | 17,60 | 24,10 | 20,20 | 17,90 | 15,20 | 11,00 | 8,50 |
| stds | 0,05 | 0,50 | 1,50 | 1,10 | 0,90 | 0,50 | 2,10 | 0,60 | 0,00 |
| rstds | 0,01 | 0,05 | 0,09 | 0,05 | 0,04 | 0,03 | 0,14 | 0,05 | 0,00 |
