# Supplementary material for: Profiles of Endogenous Phytohormones Over the Course of Norway Spruce Somatic Embryogenesis
Source: Front Plant Sci. 2018 Sep 6;9:1283. doi: 10.3389/fpls.2018.01283 (PMC6136392; doi:10.3389/fpls.2018.01283)
Supplement: TABLE S2 — Complete list of the distribution and endogenous levels (pmol/g DW) of phytohormones and their derivatives in the P. abies embryogenic cultures during the process of somatic embryogenesis. [file Table_2.DOCX]

**Table S2. Complete list of the distribution and endogenous levels (pmol/g DW) of phytohormones and their derivatives in the *Picea abies* embryogenic cultures during the process of somatic embryogenesis.**

Mean values (A) of five independent measurements are shown, including standard deviations (stds) and relative standard deviations (rstds). Regarding the specificity of plant material values, only data points with rstds < 0.35 were considered reliable (95 % of the total); data points with rstds > 0.35 (5 % of the total) are designated by grey boxes.

|  |  | **P** | **M3** | **M5** | **D1** | **D2** | **D3** | **G1** | **G4** | **G7** |
| --- | --- | --- | --- | --- | --- | --- | --- | --- | --- | --- |
| ***AUXINS*** |  |  |  |  |  |  |  |  |  |  |
| IAA | A | 308,87 | 1531,57 | 718,98 | 340,84 | 424,61 | 401,52 | 351,01 | 350,26 | 368,34 |
|  | stds | 38,95 | 370,06 | 65,40 | 29,07 | 65,15 | 39,55 | 25,17 | 39,71 | 59,04 |
|  | rstds | 0,13 | 0,24 | 0,09 | 0,09 | 0,15 | 0,10 | 0,07 | 0,11 | 0,16 |
|  |  |  |  |  |  |  |  |  |  |  |
| IAA-Asp | A | 59,42 | 8713,79 | 307,19 | 776,34 | 419,71 | 251,13 | 209,53 | 207,28 | 91,78 |
|  | stds | 7,32 | 639,01 | 42,13 | 43,54 | 102,40 | 44,77 | 40,80 | 50,89 | 14,46 |
|  | rstds | 0,12 | 0,07 | 0,14 | 0,06 | 0,24 | 0,18 | 0,19 | 0,25 | 0,16 |
|  |  |  |  |  |  |  |  |  |  |  |
| IAA-Glu | A | 15,12 | 7577,63 | 283,66 | 463,91 | 135,58 | 106,74 | 59,18 | 63,58 | 51,24 |
|  | stds | 1,46 | 1654,85 | 58,03 | 100,96 | 28,20 | 15,86 | 16,42 | 14,88 | 3,55 |
|  | rstds | 0,10 | 0,22 | 0,20 | 0,22 | 0,21 | 0,15 | 0,28 | 0,23 | 0,07 |
|  |  |  |  |  |  |  |  |  |  |  |
| OxIAA | A | 507,49 | 280,21 | 309,69 | 482,70 | 769,79 | 921,07 | 250,18 | 166,61 | 166,20 |
|  | stds | 94,35 | 77,85 | 75,35 | 82,20 | 214,56 | 95,18 | 65,79 | 23,79 | 40,93 |
|  | rstds | 0,19 | 0,28 | 0,24 | 0,17 | 0,28 | 0,10 | 0,26 | 0,14 | 0,25 |
|  |  |  |  |  |  |  |  |  |  |  |
| OxIAA-GE | A | 9,23 | 11,19 | 0,43 | 19,06 | 120,98 | 66,85 | 6,77 | 60,93 | 6,09 |
|  | stds | 2,84 | 2,57 | 0,08 | 4,57 | 9,83 | 18,17 | 1,85 | 12,40 | 2,95 |
|  | rstds | 0,31 | 0,23 | 0,18 | 0,24 | 0,08 | 0,27 | 0,27 | 0,20 | 0,48 |
|  |  |  |  |  |  |  |  |  |  |  |
| PAA | A | 4531,95 | 10957,69 | 3221,46 | 1178,82 | 1966,91 | 2399,28 | 4143,95 | 10241,21 | 12644,55 |
|  | stds | 978,89 | 2212,03 | 988,08 | 113,15 | 482,98 | 542,05 | 81,05 | 2559,61 | 2214,47 |
|  | rstds | 0,22 | 0,20 | 0,31 | 0,10 | 0,25 | 0,23 | 0,02 | 0,25 | 0,18 |
|  |  |  |  |  |  |  |  |  |  |  |
| **Auxins total** | A | 5432,08 | 29072,09 | 4841,42 | 3261,67 | 3837,58 | 4146,58 | 5020,62 | 11089,88 | 13328,20 |
|  | stds | 984,23 | 2860,59 | 995,69 | 180,32 | 543,07 | 554,10 | 116,05 | 2560,61 | 2215,69 |
|  | rstds | 0,18 | 0,10 | 0,21 | 0,06 | 0,14 | 0,13 | 0,02 | 0,23 | 0,17 |
|  |  |  |  |  |  |  |  |  |  |  |
| ***CYTOKININS*** |  |  |  |  |  |  |  |  |  |  |
| *cisZ* | A | 26,62 | 5,13 | 6,16 | 4,35 | 4,19 | 6,20 | 8,95 | 10,86 | 10,08 |
|  | stds | 4,74 | 0,57 | 0,98 | 1,58 | 0,23 | 2,02 | 1,53 | 1,05 | 3,09 |
|  | rstds | 0,18 | 0,11 | 0,16 | 0,36 | 0,06 | 0,33 | 0,17 | 0,10 | 0,31 |
|  |  |  |  |  |  |  |  |  |  |  |
| *cis*ZR | A | 11,44 | 21,75 | 11,65 | 12,42 | 15,50 | 18,60 | 21,97 | 26,94 | 35,10 |
|  | stds | 1,15 | 0,95 | 0,70 | 3,20 | 2,48 | 1,05 | 5,22 | 4,11 | 3,84 |
|  | rstds | 0,10 | 0,04 | 0,06 | 0,26 | 0,16 | 0,06 | 0,24 | 0,15 | 0,11 |
|  |  |  |  |  |  |  |  |  |  |  |
| *c*isZOG | A | 0,17 | 0,35 | 3,44 | 8,42 | 5,51 | 33,19 | 26,66 | 38,60 | 40,29 |
|  | stds | 0,01 | 0,00 | 0,31 | 0,40 | 0,29 | 3,76 | 2,11 | 2,16 | 6,04 |
|  | rstds | 0,05 | 0,01 | 0,09 | 0,05 | 0,05 | 0,11 | 0,08 | 0,06 | 0,15 |
|  |  |  |  |  |  |  |  |  |  |  |
| *cis*ZROG | A | 93,70 | 17,27 | 39,51 | 162,64 | 259,11 | 324,17 | 246,88 | 294,01 | 279,54 |
|  | stds | 3,62 | 15,40 | 3,40 | 23,95 | 6,65 | 41,87 | 20,67 | 24,91 | 45,77 |
|  | rstds | 0,04 | 0,89 | 0,09 | 0,15 | 0,03 | 0,13 | 0,08 | 0,08 | 0,16 |
|  |  |  |  |  |  |  |  |  |  |  |
| *cis*ZRMP | A | 10,04 | 114,71 | 90,93 | 263,01 | 227,86 | 164,77 | 93,31 | 99,22 | 71,89 |
|  | stds | 1,74 | 15,41 | 18,95 | 12,80 | 54,97 | 12,91 | 21,63 | 8,63 | 12,55 |
|  | rstds | 0,17 | 0,13 | 0,21 | 0,05 | 0,24 | 0,08 | 0,23 | 0,09 | 0,17 |
|  |  |  |  |  |  |  |  |  |  |  |
| DHZ | A | 3,41 | 23,67 | 24,83 | 12,84 | 6,90 | 8,87 | 7,39 | 5,89 | 8,09 |
|  | stds | 0,17 | 12,95 | 0,81 | 0,08 | 0,86 | 0,70 | 2,32 | 0,52 | 2,20 |
|  | rstds | 0,05 | 0,55 | 0,03 | 0,01 | 0,13 | 0,08 | 0,31 | 0,09 | 0,27 |
|  |  |  |  |  |  |  |  |  |  |  |
| DHZR | A | 8,48 | 15,81 | 17,76 | 5,68 | 9,74 | 9,04 | 7,26 | 54,93 | 229,03 |
|  | stds | 0,98 | 1,03 | 1,65 | 1,54 | 3,78 | 0,44 | 1,17 | 18,04 | 94,20 |
|  | rstds | 0,12 | 0,06 | 0,09 | 0,27 | 0,39 | 0,05 | 0,16 | 0,33 | 0,41 |
|  |  |  |  |  |  |  |  |  |  |  |
| DHZROG | A | 214,25 | 135,27 | 116,50 | 342,06 | 239,04 | 83,41 | 95,27 | 75,40 | 129,37 |
|  | stds | 31,17 | 41,36 | 9,52 | 2,28 | 43,08 | 9,76 | 8,71 | 4,11 | 36,96 |
|  | rstds | 0,15 | 0,31 | 0,08 | 0,01 | 0,18 | 0,12 | 0,09 | 0,05 | 0,29 |
|  |  |  |  |  |  |  |  |  |  |  |
| DHZRMP | A | 2,58 | 22,91 | 33,87 | 23,69 | 25,47 | 18,21 | 7,14 | 5,56 | 3,12 |
|  | stds | 0,54 | 4,38 | 9,49 | 6,81 | 4,65 | 3,34 | 1,57 | 1,67 | 0,72 |
|  | rstds | 0,21 | 0,19 | 0,28 | 0,29 | 0,18 | 0,18 | 0,22 | 0,30 | 0,23 |
|  |  |  |  |  |  |  |  |  |  |  |
| *trans*Z | A | 5,64 | 14,14 | 46,94 | 2,23 | 3,62 | 26,13 | 3,88 | 10,60 | 3,21 |
|  | stds | 0,31 | 9,43 | 10,07 | 0,51 | 0,85 | 8,15 | 0,84 | 6,24 | 0,93 |
|  | rstds | 0,06 | 0,67 | 0,21 | 0,23 | 0,24 | 0,31 | 0,22 | 0,59 | 0,29 |
|  |  |  |  |  |  |  |  |  |  |  |
| *trans*ZR | A | 9,46 | 4,31 | 4,85 | 2,03 | 4,57 | 0,36 | 8,81 | 16,84 | 54,02 |
|  | stds | 2,23 | 0,61 | 0,89 | 0,04 | 1,42 | 0,01 | 10,80 | 3,67 | 28,28 |
|  | rstds | 0,24 | 0,14 | 0,18 | 0,02 | 0,31 | 0,03 | 1,23 | 0,22 | 0,52 |
|  |  |  |  |  |  |  |  |  |  |  |
| *trans*ZOG | A | 5,14 | 5,07 | 2,09 | 3,90 | 4,37 | 2,86 | 3,53 | 3,15 | 6,83 |
|  | stds | 0,74 | 0,19 | 0,24 | 1,56 | 1,00 | 0,26 | 0,00 | 0,94 | 0,60 |
|  | rstds | 0,14 | 0,04 | 0,12 | 0,40 | 0,23 | 0,09 | 0,00 | 0,30 | 0,09 |
|  |  |  |  |  |  |  |  |  |  |  |
| *trans*ZROG | A | 1,29 | 20,97 | 4,46 | 8,18 | 15,17 | 12,29 | 5,27 | 13,25 | 119,53 |
|  | stds | 0,06 | 11,23 | 0,56 | 0,81 | 0,77 | 2,11 | 1,01 | 5,84 | 34,01 |
|  | rstds | 0,04 | 0,54 | 0,13 | 0,10 | 0,05 | 0,17 | 0,19 | 0,44 | 0,28 |
|  |  |  |  |  |  |  |  |  |  |  |
| *trans*ZRMP | A | 13,61 | 34,65 | 13,27 | 8,78 | 6,03 | 1,54 | 2,68 | 7,14 | 7,47 |
|  | stds | 1,96 | 4,51 | 3,35 | 1,94 | 1,14 | 0,11 | 0,12 | 1,47 | 1,49 |
|  | rstds | 0,14 | 0,13 | 0,25 | 0,22 | 0,19 | 0,07 | 0,04 | 0,21 | 0,20 |
|  |  |  |  |  |  |  |  |  |  |  |
| iP | A | 7,67 | 8,13 | 4,04 | 2,79 | 6,42 | 3,59 | 4,99 | 5,28 | 16,87 |
|  | stds | 0,15 | 2,87 | 0,67 | 0,41 | 0,06 | 0,46 | 0,67 | 0,85 | 2,57 |
|  | rstds | 0,02 | 0,35 | 0,17 | 0,15 | 0,01 | 0,13 | 0,13 | 0,16 | 0,15 |
|  |  |  |  |  |  |  |  |  |  |  |
| iPR | A | 1,29 | 2,07 | 4,10 | 6,34 | 6,75 | 2,63 | 18,91 | 10,05 | 61,08 |
|  | stds | 0,18 | 0,64 | 1,16 | 1,85 | 3,41 | 0,16 | 0,65 | 0,05 | 19,32 |
|  | rstds | 0,14 | 0,31 | 0,28 | 0,29 | 0,51 | 0,06 | 0,03 | 0,00 | 0,32 |
|  |  |  |  |  |  |  |  |  |  |  |
| iPRMP | A | 23,85 | 23,05 | 71,61 | 86,81 | 73,37 | 55,66 | 138,52 | 103,39 | 113,13 |
|  | stds | 2,09 | 5,26 | 10,44 | 13,79 | 11,97 | 1,71 | 31,70 | 14,16 | 20,05 |
|  | rstds | 0,09 | 0,23 | 0,15 | 0,16 | 0,16 | 0,03 | 0,23 | 0,14 | 0,18 |
|  |  |  |  |  |  |  |  |  |  |  |
| **Cytokinins total** | A | 438,64 | 469,27 | 496,02 | 956,16 | 913,62 | 771,52 | 701,41 | 781,11 | 1188,65 |
|  | stds | 32,04 | 51,44 | 27,95 | 31,7 | 71,59 | 46,05 | 46,25 | 36,79 | 123,69 |
|  | rstds | 0,07 | 0,11 | 0,06 | 0,03 | 0,09 | 0,06 | 0,07 | 0,05 | 0,10 |
|  |  |  |  |  |  |  |  |  |  |  |
| ***ABSCISIC ACID + DERIVATIVES*** |  |  |  |  |  |  |  |  |  |  |
| ABA | A | 142,36 | 540067,27 | 349804,48 | 164116,26 | 184251,32 | 178750,51 | 59571,82 | 9639,03 | 6932,86 |
|  | stds | 17,01 | 85494,75 | 55046,62 | 7939,75 | 30573,20 | 14985,54 | 1864,75 | 1773,78 | 666,70 |
|  | rstds | 0,12 | 0,16 | 0,16 | 0,05 | 0,17 | 0,08 | 0,03 | 0,18 | 0,10 |
|  |  |  |  |  |  |  |  |  |  |  |
| DPA | A | 11,36 | 210252,70 | 84314,11 | 2951,33 | 3692,83 | 4079,39 | 2526,39 | 1018,62 | 346,07 |
|  | stds | 2,03 | 31521,98 | 9465,38 | 759,26 | 483,90 | 764,09 | 484,90 | 131,24 | 11,56 |
|  | rstds | 0,18 | 0,15 | 0,11 | 0,26 | 0,13 | 0,19 | 0,19 | 0,13 | 0,03 |
|  |  |  |  |  |  |  |  |  |  |  |
| PA | A | 15,42 | 36638,62 | 16225,03 | 175,13 | 211,67 | 171,71 | 331,32 | 60,43 | 34,13 |
|  | stds | 3,95 | 7764,67 | 2091,93 | 17,76 | 55,95 | 32,05 | 59,25 | 14,16 | 4,22 |
|  | rstds | 0,26 | 0,21 | 0,13 | 0,10 | 0,26 | 0,19 | 0,18 | 0,23 | 0,12 |
|  |  |  |  |  |  |  |  |  |  |  |
| ABA-GE | A | 131,19 | 14128,32 | 48115,03 | 47515,12 | 89145,34 | 36287,39 | 52393,62 | 27037,40 | 14199,84 |
|  | stds | 12,72 | 8716,57 | 9249,12 | 8820,65 | 6721,28 | 9930,21 | 11715,83 | 5301,66 | 3732,36 |
|  | rstds | 0,10 | 0,62 | 0,19 | 0,19 | 0,08 | 0,27 | 0,22 | 0,20 | 0,26 |
|  |  |  |  |  |  |  |  |  |  |  |
| NeoPA | A | 1,50 | 1188,85 | 551,95 | 30,23 | 30,57 | 11,51 | 7,66 | 4,35 | 1,66 |
|  | stds | 0,39 | 214,30 | 66,10 | 8,47 | 9,14 | 3,61 | 0,08 | 0,61 | 0,04 |
|  | rstds | 0,26 | 0,18 | 0,12 | 0,28 | 0,30 | 0,31 | 0,01 | 0,14 | 0,03 |
|  |  |  |  |  |  |  |  |  |  |  |
| 9-OH ABA | A | 3,04 | 25303,22 | 4983,79 | 87,96 | 86,01 | 83,53 | 68,85 | 23,28 | 10,73 |
|  | stds | 0,63 | 3025,04 | 387,85 | 15,20 | 10,91 | 10,98 | 4,63 | 3,69 | 3,79 |
|  | rstds | 0,21 | 0,12 | 0,08 | 0,17 | 0,13 | 0,13 | 0,07 | 0,16 | 0,35 |
|  |  |  |  |  |  |  |  |  |  |  |
| **Abscisic acid + derivatives total** | A | 304,87 | 827578,98 | 503994,38 | 214876,02 | 277417,74 | 219384,05 | 114899,65 | 37783,10 | 21525,29 |
|  | stds | 21,71 | 91915,47 | 56655,11 | 11892,04 | 31307,09 | 17993,35 | 11873,36 | 5592,07 | 3791,46 |
|  | rstds | 0,07 | 0,11 | 0,11 | 0,06 | 0,11 | 0,08 | 0,10 | 0,15 | 0,18 |
|  |  |  |  |  |  |  |  |  |  |  |
| ***JASMONATES*** |  |  |  |  |  |  |  |  |  |  |
| JA | A | 139,39 | 266,39 | 196,37 | 101,43 | 326,63 | 525,25 | 1342,59 | 2964,87 | 1686,29 |
|  | stds | 51,02 | 31,48 | 41,46 | 4,78 | 59,86 | 44,59 | 201,00 | 229,97 | 137,36 |
|  | rstds | 0,37 | 0,12 | 0,21 | 0,05 | 0,18 | 0,08 | 0,15 | 0,08 | 0,08 |
|  |  |  |  |  |  |  |  |  |  |  |
| JA Ileu | A | 180,55 | 32,63 | 6,28 | 18,74 | 8,33 | 40,03 | 161,06 | 124,50 | 356,63 |
|  | stds | 45,42 | 0,37 | 1,12 | 5,30 | 1,69 | 10,61 | 8,52 | 45,09 | 70,15 |
|  | rstds | 0,25 | 0,01 | 0,18 | 0,28 | 0,20 | 0,26 | 0,05 | 0,36 | 0,20 |
|  |  |  |  |  |  |  |  |  |  |  |
| cisOPDA | A | 86,93 | 307,59 | 309,59 | 185,20 | 508,60 | 428,31 | 944,39 | 2949,54 | 6276,69 |
|  | stds | 8,85 | 5,01 | 111,40 | 41,03 | 41,04 | 76,17 | 47,11 | 163,49 | 268,28 |
|  | rstds | 0,10 | 0,02 | 0,36 | 0,22 | 0,08 | 0,18 | 0,05 | 0,06 | 0,04 |
|  |  |  |  |  |  |  |  |  |  |  |
| **Jasmonates total** | A | 406,87 | 606,61 | 512,24 | 305,37 | 843,57 | 993,60 | 2448,04 | 6038,91 | 8319,62 |
|  | stds | 68,88 | 31,88 | 118,87 | 41,65 | 72,60 | 88,90 | 206,62 | 285,74 | 309,46 |
|  | rstds | 0,17 | 0,05 | 0,23 | 0,14 | 0,09 | 0,09 | 0,08 | 0,05 | 0,04 |
|  |  |  |  |  |  |  |  |  |  |  |
| ***SALICYLIC and BENZOIC ACIDS*** | | |  |  |  |  |  |  |  |  |
| SA | A | 20869,21 | 6330,56 | 2935,06 | 2479,56 | 1211,94 | 1238,82 | 1283,58 | 1947,73 | 3413,26 |
|  | stds | 150,70 | 884,25 | 355,99 | 505,59 | 78,42 | 135,71 | 260,40 | 460,81 | 716,64 |
|  | rstds | 0,01 | 0,14 | 0,12 | 0,20 | 0,06 | 0,11 | 0,20 | 0,24 | 0,21 |
|  |  |  |  |  |  |  |  |  |  |  |
| BzA | A | 19891,42 | 67153,70 | 19737,63 | 16356,46 | 24049,95 | 15531,28 | 14230,71 | 25701,85 | 30851,96 |
|  | stds | 9205,03 | 3739,53 | 2252,68 | 3092,91 | 1113,40 | 2956,67 | 2597,81 | 7786,46 | 5351,05 |
|  | rstds | 0,46 | 0,06 | 0,11 | 0,19 | 0,05 | 0,19 | 0,18 | 0,30 | 0,17 |
