# Supplementary material for: Profiles of Endogenous Phytohormones Over the Course of Norway Spruce Somatic Embryogenesis
Source: Front Plant Sci. 2018 Sep 6;9:1283. doi: 10.3389/fpls.2018.01283 (PMC6136392; doi:10.3389/fpls.2018.01283)
Supplement: TABLE S3 — Principal components analysis (PCA) summarizing correlations between individual embryo developmental stages and the dynamics of the total concentrations of four particular phytohormone groups (auxins, CKs, ABA, and jasmonates) and two individual phytohormone species (salicylic and benzoic acids) in the P. abies embryogenic cultures. [file Table_3.DOCX]

**Table S3. Principal components analysis (PCA) summarizing correlations between individual embryo developmental stages and the dynamics of the total concentrations of four particular phytohormone groups (auxins, cytokinins, abscisic acid and jasmonates) and two individual phytohormone species (salicylic and benzoic acids) in the *Picea abies* embryogenic cultures.**

The PCA was performed on the correlation matrix to offset the large difference in the scales of the variables. The first two components of PCA were considered. The Microcal Origin Pro 2018 statistical package was used.

|  | Eigenvalues of the correlation matrix | | |
| --- | --- | --- | --- |
|  |  |  |  |
|  | **Eigenvalue** | **Percentage of Variance** | **Cumulative** |
| 1 | 2,79995 | 46,67% | 46,67% |
| 2 | 1,78898 | 29,82% | 76,48% |
| 3 | 1,08746 | 18,12% | 94,61% |
| 4 | 0,27116 | 4,52% | 99,13% |
| 5 | 0,04482 | 0,75% | 99,87% |
| 6 | 0,00764 | 0,13% | 100,00% |
|  |  |  |  |

| Correlation matrix | |  |  |  |  |  |
| --- | --- | --- | --- | --- | --- | --- |
|  |  |  |  |  |  |  |
|  | **Auxins** | **Cytokinins** | **Abscisic acid** | **Jasmonates** | **SA** | **BzA** |
| **Auxins** | 1,0000 | -0,2349 | 0,5992 | 0,2000 | 0,0766 | 0,9705 |
| **Cytokinins** | 0,2349 | 1,0000 | -0,4666 | 0,5845 | -0,5371 | -0,2589 |
| **Abscisic acid** | 0,5992 | -0,4666 | 1,0000 | -0,4919 | -0,1563 | 0,7064 |
| **Jasmonates** | 0,2000 | 0,5845 | -0,4919 | 1,0000 | -0,2371 | 0,0399 |
| **Salicylic acid** | 0,0766 | -0,5371 | -0,1563 | -0,2371 | 1,0000 | 0,0954 |
| **Benzoic acid** | 0,9705 | -0,2589 | 0,7064 | 0,0399 | 0,0954 | 1,0000 |

| Factor loadings | |  |  |  |
| --- | --- | --- | --- | --- |
|  |  |  |  |  |
|  | **PC1** | **PC2** | **PC3** | **PC4** |
| **Auxins** | 0,4868 | 0,3885 | 0,2271 | -0,0280 |
| **Cytokinins** | 0,3889 | 0,4763 | -0,1170 | 0,7562 |
| **Abscisic acid** | 0,5160 | 0,0309 | -0,4605 | 0,0321 |
| **Jasmonates** | 0,2212 | 0,5767 | 0,4283 | -0,4662 |
| **Salicylic acid** | 0,1489 | -0,4264 | 0,7226 | 0,3827 |
| **Benzoic acid** | 0,5239 | 0,3268 | 0,1312 | 0,2501 |
