# Supplementary figures and images for: Profiles of Endogenous Phytohormones Over the Course of Norway Spruce Somatic Embryogenesis
Source: Front Plant Sci. 2018 Sep 6;9:1283. doi: 10.3389/fpls.2018.01283 (PMC6136392; doi:10.3389/fpls.2018.01283)

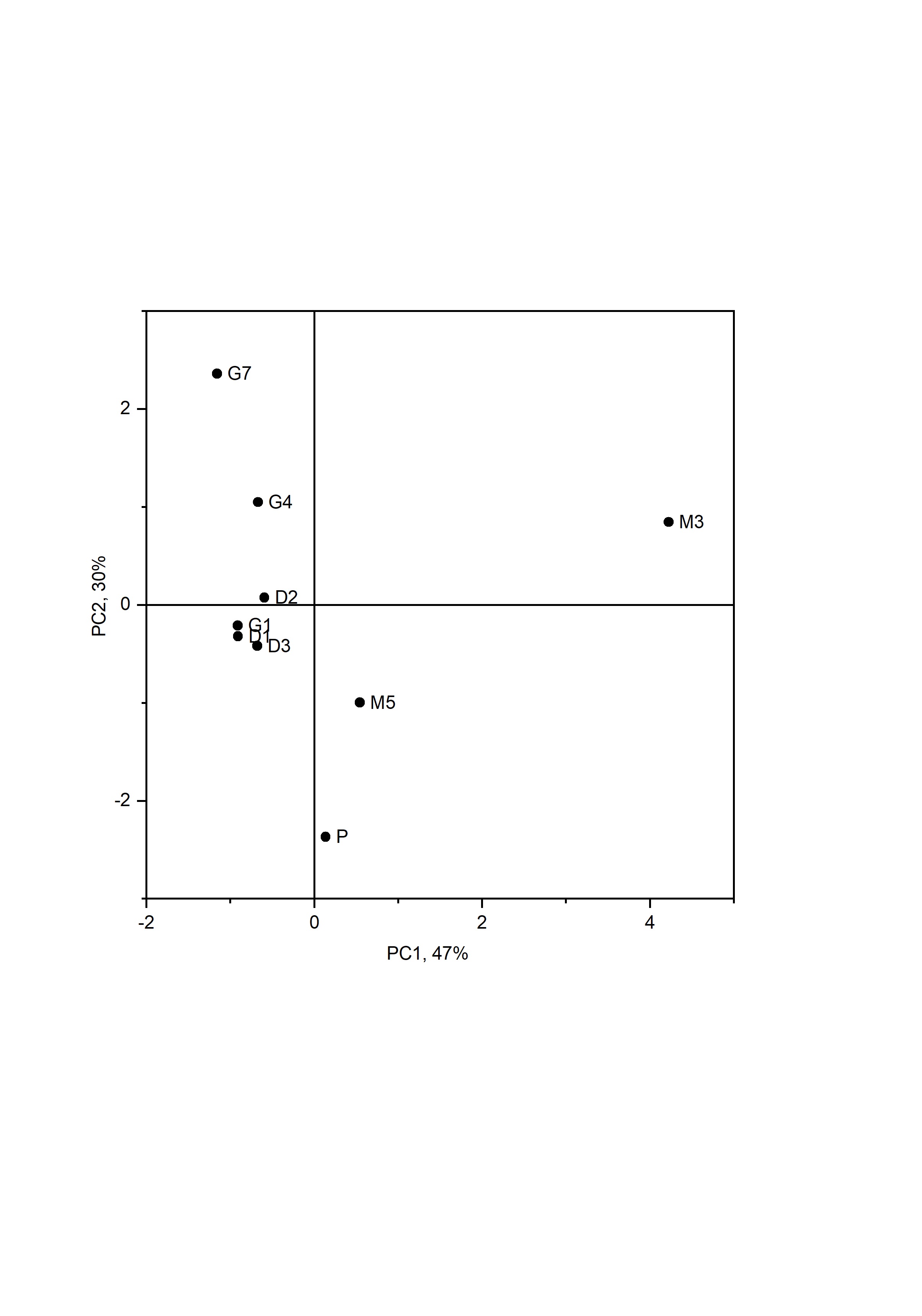

Supplement: FIGURE S1 — Principal component analysis (PCA) space of the phytohormone concentrations examined, defined by the first two components (PC1 and PC2) for all embryo developmental stages in the P. abies embryogenic cultures. P, proliferation; M, maturation (M3, M5 – 3 and 5 weeks of maturation, respectively); D, desiccation (D1, D2, D3 – 1, 2, and 3 weeks of desiccation, respectively); G, germination (G1, G4, G7 – 1, 4, and 7 days of germination, respectively). [file Image_1.JPEG]
